# Malignant solitary fibrous tumor in the anterior abdominal wall: a rare case report with diagnostic and therapeutic insights

**DOI:** 10.1093/jscr/rjaf289

**Published:** 2025-05-08

**Authors:** Abdul Muqeet, Danish Hassan, Emad Uddin Sajid, Absar Hyder, Muhammad Imran Siraj, Faisal Ibrahim

**Affiliations:** Dow University of Health Sciences, Karachi, Pakistan; Dow University of Health Sciences, Karachi, Pakistan; Dow University of Health Sciences, Karachi, Pakistan; Dow University of Health Sciences, Karachi, Pakistan; Dow University of Health Sciences, Karachi, Pakistan; Dow University of Health Sciences, Karachi, Pakistan

**Keywords:** solitary fibrous tumor, anterior abdominal wall muscle, left iliac fossa mass, abdominal mass

## Abstract

Solitary fibrous tumors (SFTs) are rare mesenchymal tumors, most commonly occurring in the pleura. Extrapleural cases, particularly in the anterior abdominal wall, are extremely rare. We present a rare case of a malignant SFT arising from the left anterior abdominal wall in an adult patient in their 60s, who presented with a mass in left iliac fossa. The diagnosis was confirmed by tissue biopsy and immunohistochemical analysis. The presented case highlights the rarity of malignant SFTs in the anterior abdominal wall, emphasizing the importance of thorough diagnostic evaluation and surgical management for optimal outcomes.

## Introduction

Solitary fibrous tumor (SFT), previously known as hemangiopericytoma, is an uncommon mesenchymal neoplasm arising from CD34-positive stromal cells. While these tumors predominantly occur in the pleura, ~30% are found in extrapleural sites, such as the extremities, head and neck, and abdomen. Involvement of the abdominal wall is extremely rare, with only a few cases documented in the literature [1, 2]. According to the World Health Organization, SFTs are classified as intermediate-grade tumors with a low metastatic potential [3]. Clinical presentations vary with tumor size and location, ranging from an asymptomatic mass to abdominal pain or urinary disturbances. Imaging studies provide preliminary insights, but definitive diagnosis relies on histopathological and immunohistochemical analysis, with STAT6 nuclear staining and NAB2-STAT6 fusion gene serving as key diagnostic markers [4]. Surgical resection remains the mainstay of treatment. This report details a rare case of malignant SFT in the left iliac fossa.

## Case presentation

A 60-year-old patient with no significant medical or surgical history presented with a 10-month history of a progressively enlarging, painless mass in the left iliac fossa. The patient denied symptoms such as weight loss, fever, or bowel habit changes. Physical examination revealed a firm, non-tender swelling measuring 8 × 10 cm in the left iliac fossa. Systemic examination was unremarkable, and the abdomen was soft with normal bowel sounds.

### Investigations

#### Computed tomography chest and abdomen

**Figure 1:**
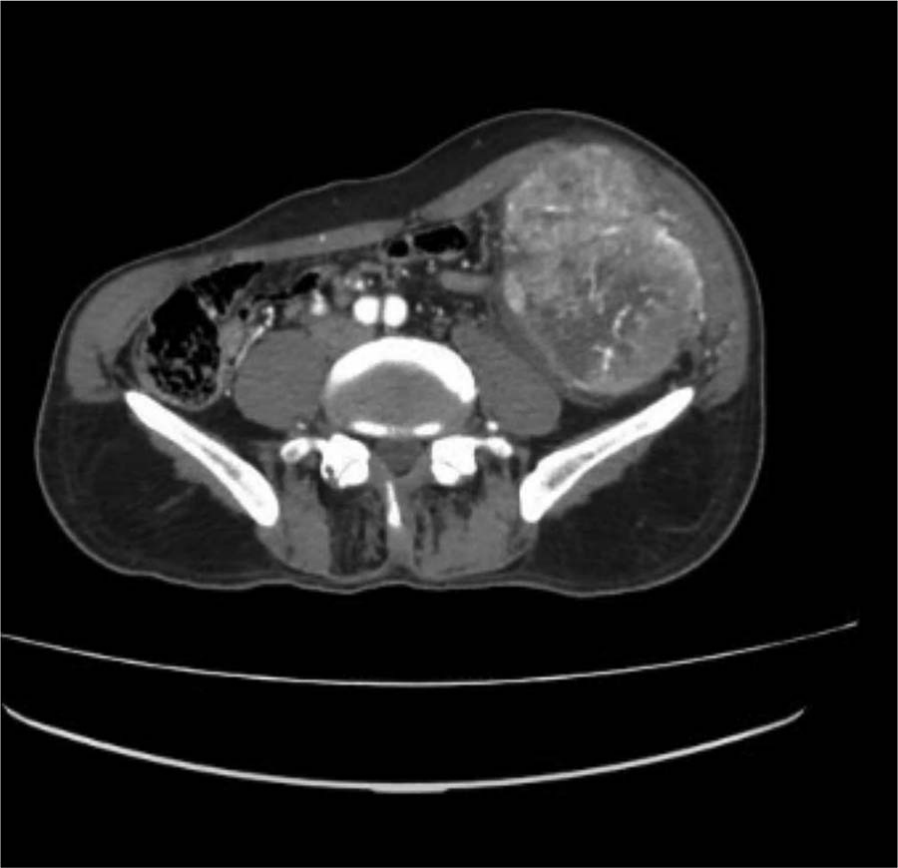
Contrast-enhanced CT scan of the abdomen showing a heterogeneously enhancing soft tissue lesion in the left iliac fossa.

A contrast-enhanced computed tomography (CT) scan of the abdomen and chest using a triphasic protocol revealed a heterogeneously enhancing soft tissue lesion in the left iliac fossa (Fig. 1), measuring 10.8 × 9.45 cm, with central necrosis and areas of calcification. The lesion displaced adjacent intestinal loops and the left psoas muscle but did not invade these structures. Tortuous veins were observed in the subcutaneous left abdominal wall, likely representing varicose veins due to venous compression. The chest was normal.

#### Biopsy results

**Figure 2:**
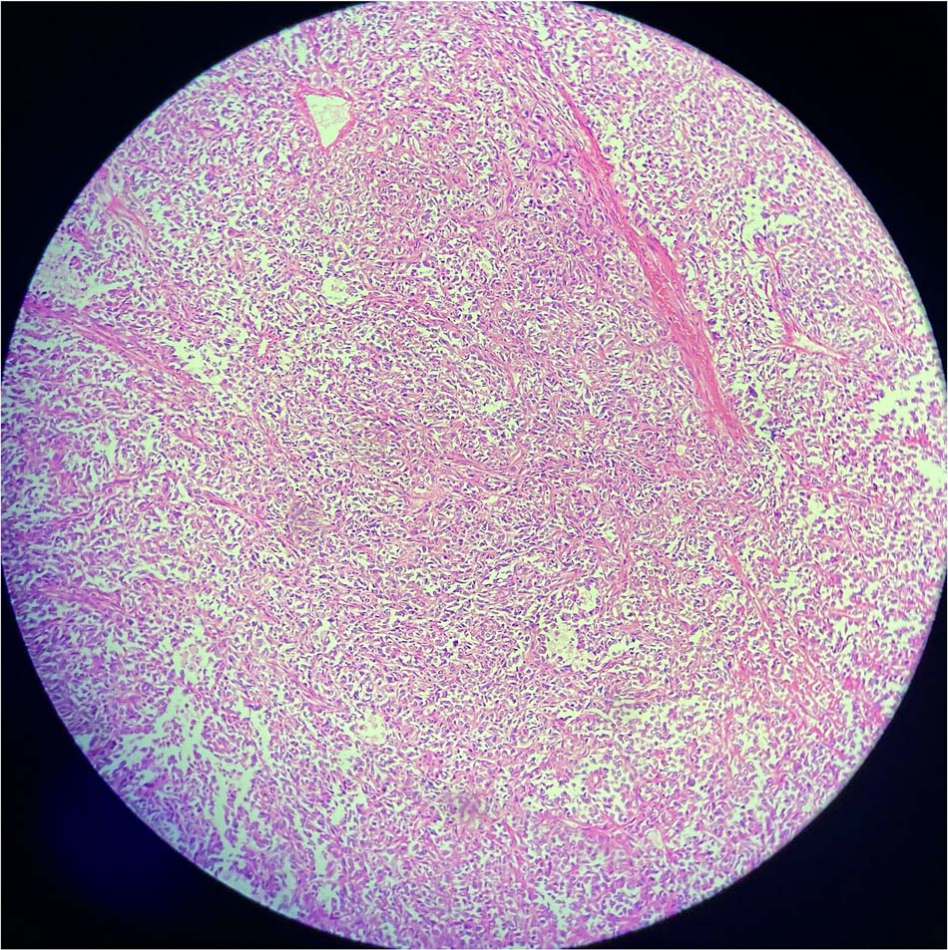
Low-power view of a solitary fibrous tumor showing a well-circumscribed lesion composed of spindle-shaped cells in a collagenous stroma. Alternating cellularity and staghorn-like branching vasculature are evident.

**Figure 3:**
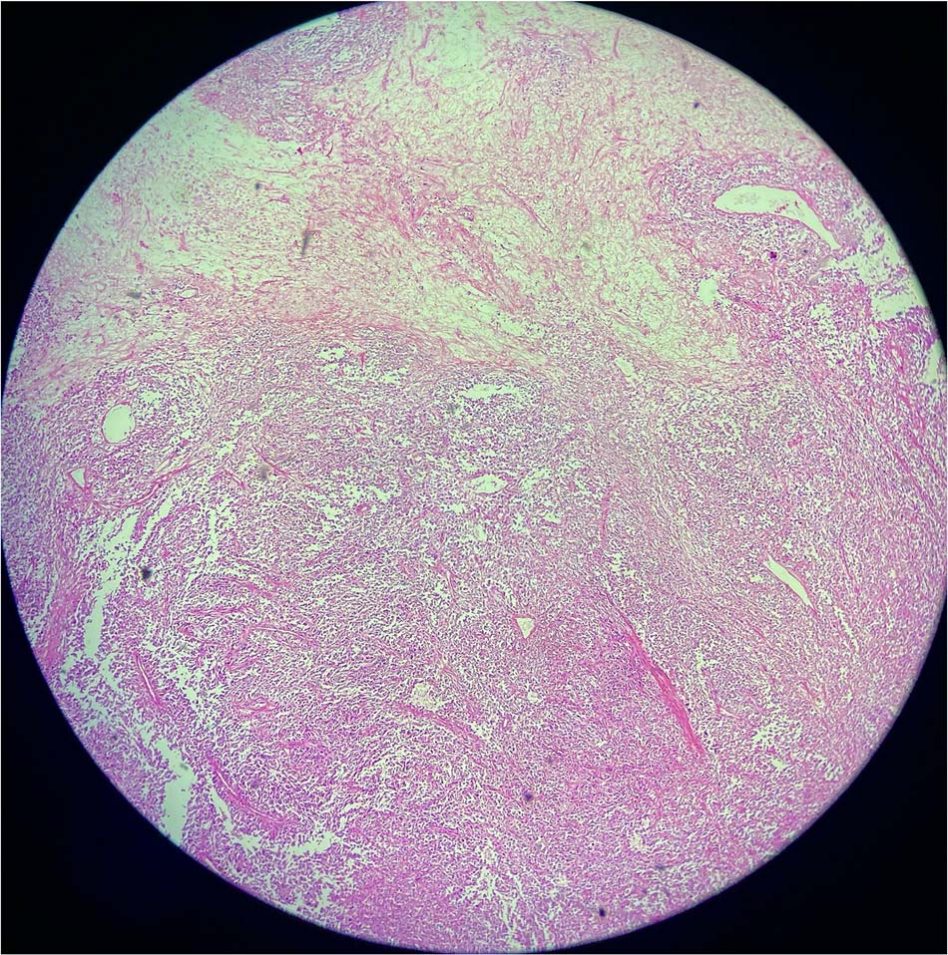
Low-power photomicrograph of a solitary fibrous tumor showing areas of tumor necrosis characterized by loss of cellular detail and eosinophilic ghost-like zones, along with alternating hypercellular and hypocellular regions and staghorn-like vasculature.

A biopsy was performed, showing malignant features consistent with SFT. Immunohistochemistry revealed positivity for CD34, STAT6, and CD99, while negative for desmin, myogenin, ASMA, cytokeratin AE1/AE3, SOX-10, EMA, TLE-1, and NKX 2.2, confirming malignant SFT (Figs 2 and 3).

#### Computed tomography angiogram

A CT angiogram demonstrated a heterogeneously enhancing solid lesion with central necrosis measuring 11.1 × 10.1 × 12.0 cm in the left iliac fossa, primarily supplied by branches from the common femoral, external iliac, and inferior mesenteric arteries. Periosteal neovascularization was noted, with patchy areas of sclerosis in the left iliac bone suggesting possible infiltration.

### Management

The patient underwent surgical excision under general anesthesia. A midline incision was made, and the tumor was dissected from surrounding structures, including the iliacus muscle, transversus abdominis muscle, and posterior rectus sheath, which were excised en bloc with the tumor. The feeding vessels were ligated, and hemostasis was achieved. A mesh was placed over the peritoneum, and histoacryl was applied. A Redivac drain was placed, and the wound was closed in layers with Vicryl sutures.

#### Post-operative care

The patient was monitored in the intensive post-operative care unit until fully awake. Postoperative medications included intravenous antibiotics (Augmentin 1.2 g every 8 h), anti-inflammatory and analgesic agents (Provas 1 g every 8 h), Toradol 30 mg IV every 8 h for pain management, and Metaclon 10 mg IV every 8 h as an antiemetic. The tumor sample was sent for histopathological examination. Drain output was monitored, and an abdominal belt was applied for additional support.

#### Follow-up and surveillance

Due to the tumor’s extensive infiltration, including suspected bony involvement, regular follow-up imaging was recommended. Contrast-enhanced magnetic resonance imaging (MRI) was scheduled every 6 months for the first 2 years, followed by annual scans for 5 years. Positron emission tomography/computed tomography would be considered for suspicious findings or recurrence.

## Discussion

SFTs are rare mesenchymal neoplasms most commonly found in the pleura, with extrapleural occurrences being significantly less frequent. Anterior abdominal wall involvement, as in this case, is exceedingly rare. Previous studies [5] have described extrapleural SFTs, emphasizing the diagnostic challenges due to their rarity and nonspecific clinical presentation. Differential diagnoses for SFTs should include other soft tissue tumors such as fibromatosis, gastrointestinal stromal tumors, and liposarcomas, which can present with similar clinical and radiological findings. Careful histopathological examination, along with immunohistochemical staining, is essential to differentiate these conditions [6]. Histologically, malignant SFTs exhibit hypercellularity, nuclear atypia, and necrosis. Immunohistochemical markers, particularly CD34 and STAT6, are crucial for diagnosis, as highlighted in studies like Chmielecki *et al*. [7], which underscore the diagnostic specificity of the NAB2-STAT6 fusion gene.

The imaging findings, including a heterogeneously enhancing lesion with central necrosis, are consistent with malignant SFTs, as described by Ginat *et al*. [8]. Alternative imaging modalities, such as MRI, may provide additional insights into the extent of local invasion, particularly for abdominal wall tumors where soft tissue involvement can be more difficult to assess on CT alone [8]. The tumor's vascular supply, identified via CT angiography, underscores its potential for local aggressiveness, including periosteal neovascularization and suspected bony infiltration. In the literature, cases of similar extrapleural SFTs have demonstrated varied clinical behavior, with some cases presenting as indolent, while others show rapid growth and invasiveness, underscoring the importance of individualized treatment approaches [9]. This case highlights the malignant potential of SFTs in extrapleural locations, where vascular features often guide surgical planning [10].

Complete surgical excision with negative margins is pivotal in managing SFTs, particularly malignant variants. Studies such as Demicco *et al*. [11] suggest that complete resection offers the best prognosis, even in malignant cases. However, alternative management options, such as radiotherapy or systemic chemotherapy, may be considered in cases where surgical resection is not feasible due to tumor size or location. These treatment options, though not standard, have been explored in limited case reports with mixed results [12]. Postoperative surveillance is crucial, given the recurrence rates of up to 30% in malignant SFTs [13]. The identification of STAT6 as a diagnostic marker not only aids in differentiation from histological mimics but also presents potential therapeutic targets. Research by Park *et al*. [14] explores the possibility of targeted therapies in refractory cases, focusing on NAB2-STAT6 fusion pathways. In comparison to other reported cases of abdominal SFTs, this case is unique in its location and malignant transformation, which may provide insight into the broader spectrum of SFT behavior in extrapleural locations. While some studies report indolent courses, this case demonstrates the aggressive nature that can occur even in unusual locations.

This case of malignant SFT in the left anterior abdominal wall underscores the importance of a comprehensive diagnostic approach and meticulous surgical management. The rarity of this tumor location necessitates heightened clinical suspicion and reliance on immunohistochemical markers like STAT6 for accurate diagnosis. Future research should focus on molecular pathways and potential targeted therapies to improve outcomes for malignant and recurrent SFTs. Regular postoperative follow-up, as implemented in this case, remains crucial for early detection of recurrence. However, this report has certain limitations. Firstly, as a single case report, the findings cannot be generalized to all patients with extrapleural malignant SFTs. Secondly, long-term follow-up data are currently lacking, and the postoperative course may evolve, requiring continued surveillance to better understand recurrence risks. Lastly, due to the nature of this case, the efficacy of adjuvant treatments like chemotherapy, radiotherapy, or targeted therapy could not be evaluated. Furthermore, molecular testing, such as detection of the NAB2-STAT6 fusion gene, was not performed in this case. While STAT6 immunohistochemistry strongly supported the diagnosis of SFT, molecular confirmation of the NAB2-STAT6 fusion could have further validated the diagnosis and provided additional insight into potential therapeutic targets.
